# Supplementary material for: Agarose-Based Hydrogel Film with Embedded Oriented Photonic Nanochains for Sensing pH
Source: Polymers (Basel). 2024 May 29;16(11):1530. doi: 10.3390/polym16111530 (PMC11174816; doi:10.3390/polym16111530)
Supplement: Supplementary file 1 [file polymers-16-01530-s001.zip › polymers-3008886-supplementary.pdf]

polymers

MDPI

Article

Agarose-Based Hydrogel Film with Embedded Oriented Photonic Nanochains for Sensing pH

Dunyi Xiao, Huiru Ma, Wei Luo and Jianguo Guan

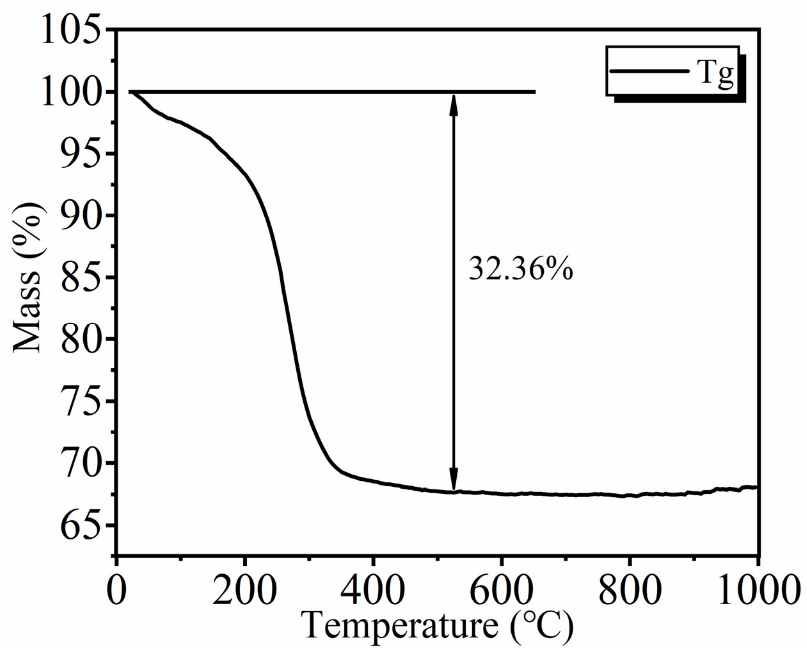

Figure S1. TG curve of the pH responsive photonic nanochains.

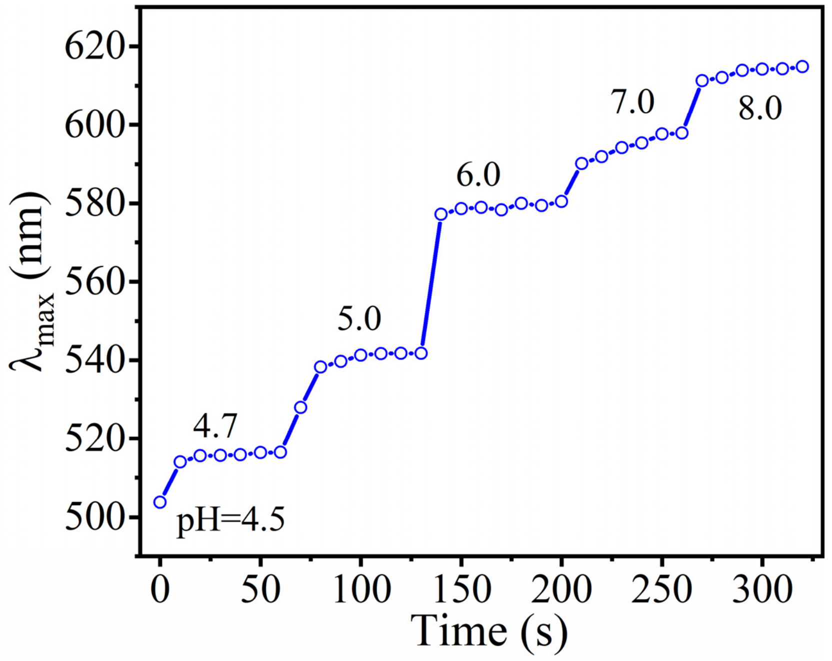

Figure S2. Response time of varying pH value.

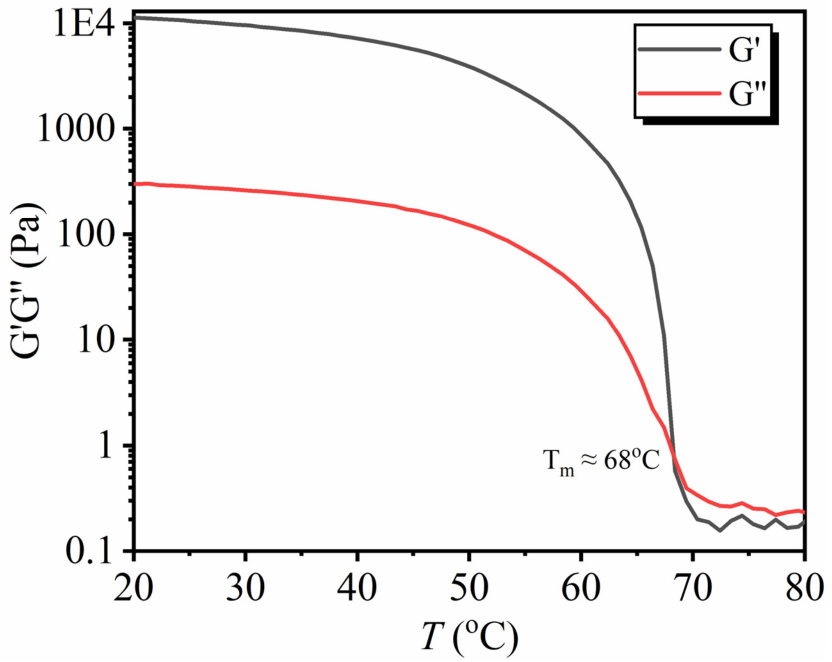

Figure S3. The variation of G' and G'' for the pH responsive heterogeneous gel film, with a shear strain set at 1%. G' and G'' respectively represent the storage modulus and the loss modulus.

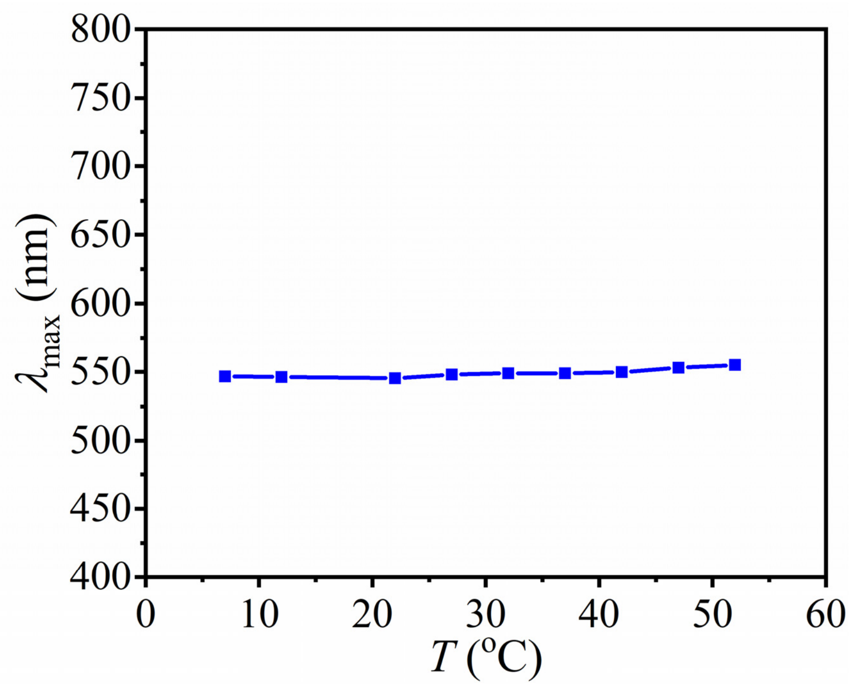

Figure S4. Dependence of λ to temperature of pH responsive heterogeneous gel film.

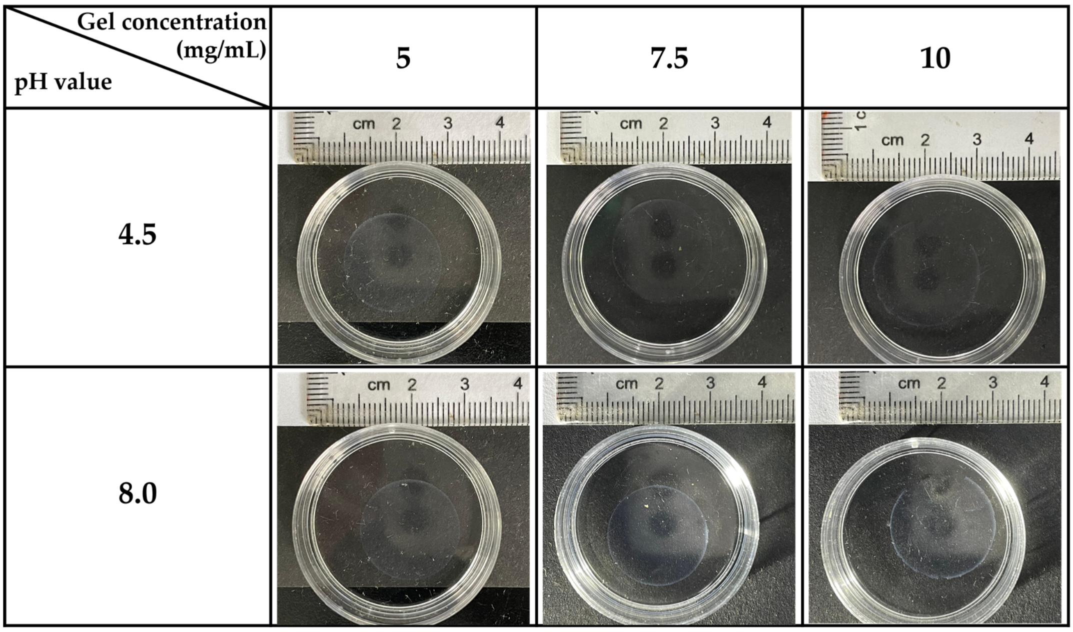

Figure S5. The digital photographs of pure gel films with agarose concentrations of 5, 7.5, and 10 mg/mL in buffer solutions with pH values of 4.5 and 8.0.

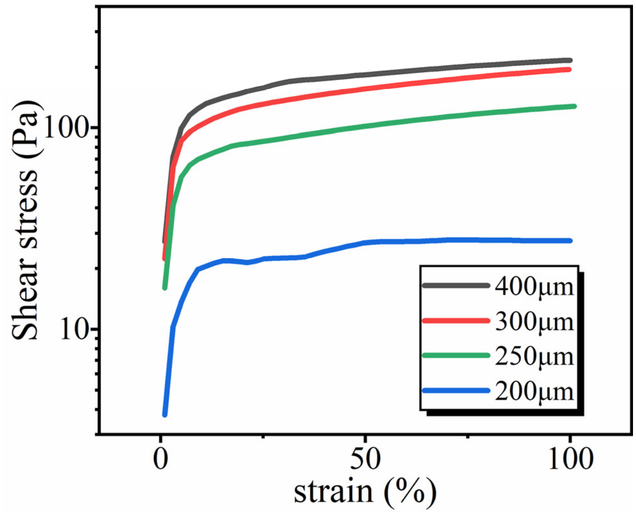

Figure S6. Shear stress-strain curves for pH-responsive heterogeneous hydrogel film with different thickness.
